# Publisher Correction: Endothelial progeria induces adipose tissue senescence and impairs insulin sensitivity through senescence associated secretory phenotype

**DOI:** 10.1038/s41467-020-17695-3

**Published:** 2020-07-28

**Authors:** Agian Jeffilano Barinda, Koji Ikeda, Dhite Bayu Nugroho, Donytra Arby Wardhana, Naoto Sasaki, Sakiko Honda, Ryota Urata, Satoaki Matoba, Ken-ichi Hirata, Noriaki Emoto

**Affiliations:** 1 Laboratory of Clinical Pharmaceutical Science, Kobe Pharmaceutical University, 4-19-1 Motoyamakitamachi, Higashinada, Kobe 658-8558, Japan; 2 Department of Pharmacology and Therapeutic, Faculty of Medicine, Universitas Indonesia, Salemba Raya 6, Jakarta, 10430 Indonesia; 3 Laboratory of Medical Pharmaceutics, Kobe Pharmaceutical University, 4-19-1 Motoyamakitamachi, Higashinada, Kobe, 658-8558 Japan; 4 Department of Cardiology, Kyoto Prefectural University Graduate School of Medical Science, 465 Kajii, Kawaramachi-Hirokoji, Kyoto, 602-8566 Japan; 5 Division of Cardiovascular Medicine, Department of Internal Medicine, Kobe University Graduate School of Medicine, 7-5-1 Kusunoki, Chuo, Kobe, 6500017 Japan

**Keywords:** Translational research, Molecular medicine

Correction to: *Nature Communications* 10.1038/s41467-020-14387-w, published online 24 January 2020.

This article contained errors in Fig. 1a and Fig. 3d. In the original version of Fig.1a, the wrong image was chosen, showing SA-β-Gal staining in 3T3-L1 adipocytes treated with conditioned medium derived from young endothelial cells (ECs) transfected with GFP. This has now been replaced with the correct image, showing SA-β-Gal staining in 3T3-L1 adipocytes treated with conditioned medium derived from young untransfected ECs. In the original version of Fig. 3d, the bottom left panel, reporting SA-β-Gal staining in 3T3-L1 adipocytes treated with the conditioned medium derived from premature senescent ECs (EC/TERF2DN) in the presence of βNMN, was inadvertently duplicated from the top left pane. This panel has now been replaced with the correct image. These errors have now been corrected in both the PDF and HTML versions of the article.

