# Supplementary material for: Endothelial progeria induces adipose tissue senescence and impairs insulin sensitivity through senescence associated secretory phenotype
Source: Nat Commun. 2020 Jan 24;11:481. doi: 10.1038/s41467-020-14387-w (PMC6981212; doi:10.1038/s41467-020-14387-w)
Supplement: Supplementary file 1 — Supplementary Information [file 41467_2020_14387_MOESM1_ESM.pdf]

Supplementary Information

Endothelial cell-specific progeria induces adipose tissue senescence and impairs insulin sensitivity through senescence-associated secretory phenotype

Barinda et al.

Supplementary Fig. 1.

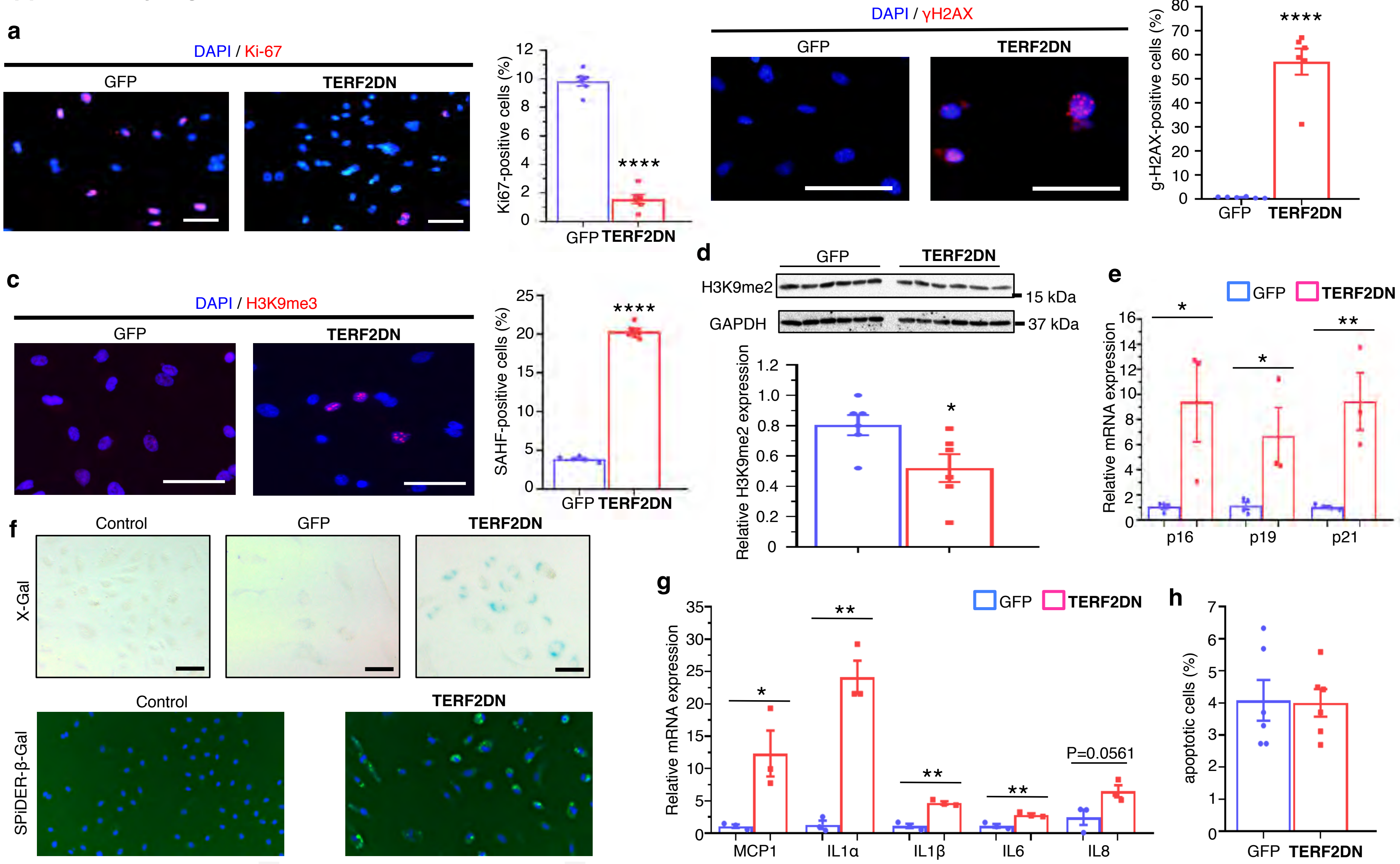

Fig. 1. Validation for cellular senescence in premature senescent EC. a, Proliferation was assessed in control (GFP) and premature senescent ECs (TERF2DN) by counting the Ki67-positive cells (n = 6 each). **b**, DNA damage was assessed by immunocytochemistry (ICC) for γH2AX. The number of γH2AX-positive nuclei was counted (n = 6 each). **c**, Senescence-associated heterochromatin foci (SAHF) was assessed by ICC for tri-methyl-histone H3 (Lys9) (H3K9me3) in control (GFP) and premature senescent ECs (TERF2DN). The number of SAHF-positive nuclei was counted (n = 5-6). **d**, Immunoblotting for histone H3 dimethyl Lys9 (H3K9me2) in control (GFP) and premature senescent ECs (TERF2DN) (n = 6 each). **e**, CDK inhibitor expression was analyzed in control (GFP) and premature senescent ECs (TERF2DN) (n = 3-4). **f**, SA-β-Gal and SPiDER-β-Gal staining in ECs without infection (control), or ECs infected with retroviruses carrying GFP or TERF2DN. **g**, SASP factor expression was analyzed in control (GFP) and premature senescent ECs (TERF2DN) (n = 3 each). **h**, Apoptotic cells were counted in control (GFP) and premature senescent ECs (TERF2DN) cultured in growth medium (n = 6 each). A two-tailed Student's *t*-test was used for statistical analysis. Data are presented as mean ± s.e. *$P < 0.05$, **$P < 0.01$, and ****$P < 0.0001$. Bars: 100 μm.

Supplementary Fig. 2.

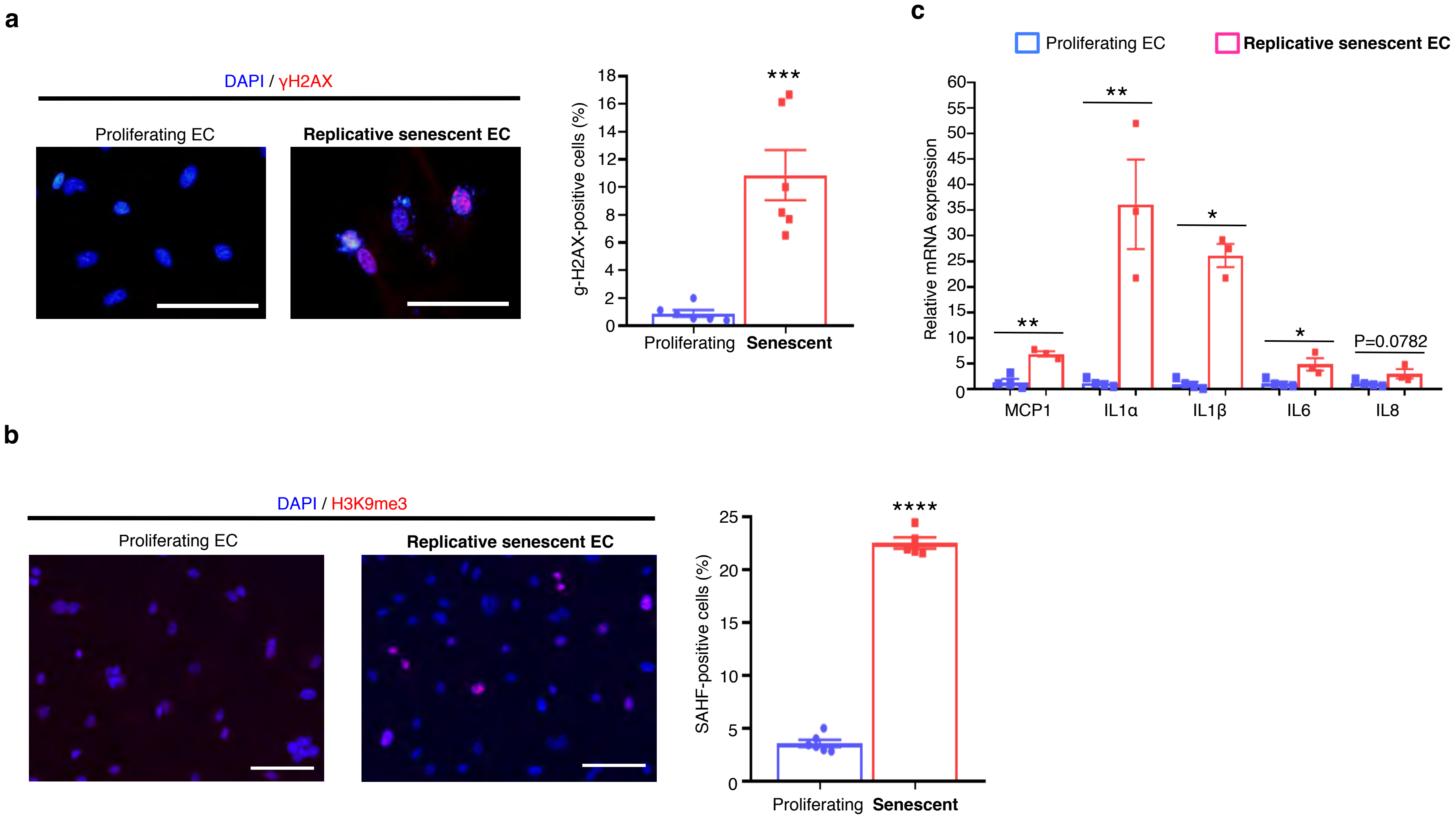

Fig. 2. Validation for cellular senescence in replicative senescent EC. a, DNA damage was assessed by ICC for γH2AX in proliferating young and replicative senescent ECs. The number of γH2AX-positive nuclei was counted (n = 6 each). **b**, SAHF was assessed by ICC for H3K9me3 in proliferating young and replicative senescent ECs. The number of SAHF-positive nuclei was counted (n = 6 for young EC; n = 5 for senescent EC). **c**, SASP factor expression was analyzed in proliferating young and replicative senescent ECs (n = 4 for young EC; n = 3 for senescent EC). A two-tailed Student's *t*-test was used for statistical analysis. Data are presented as mean ± s.e. *$P < 0.05$, **$P < 0.01$, ***$P < 0.001$, and ****$P < 0.0001$. Bars: 100 µm.

Supplementary Fig. 3.

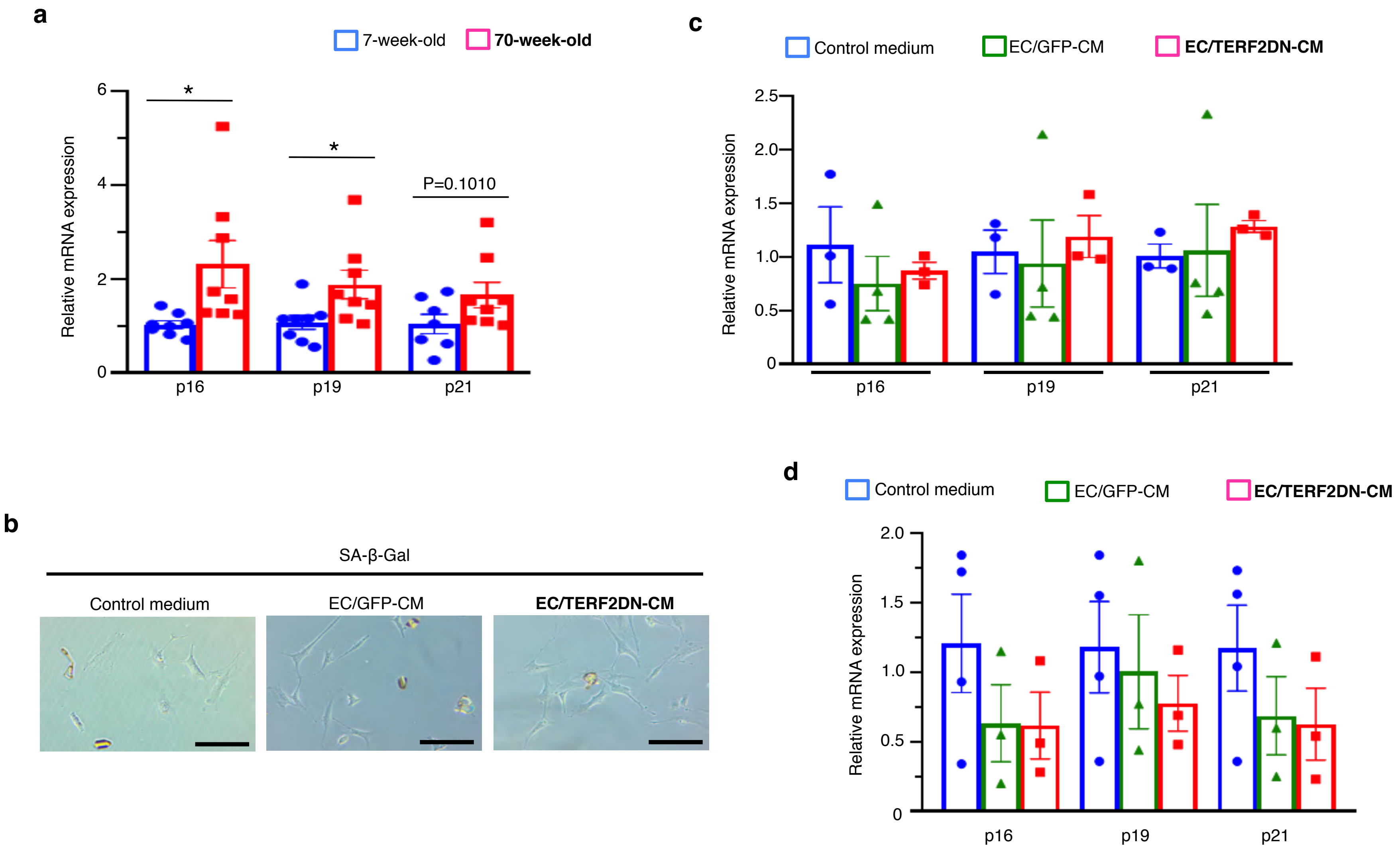

Fig. 3. Senescent EC does not induce senescence-like state in preadipocytes and myotubes. a, CDK inhibitor expression in the WAT isolated from young (7-week-old) and aged (70-week-old) mice (n = 8 each). **b**, SA-β-Gal staining in 3T3-L1 pre-adipocytes treated with the control medium, EC/GFP-CM or EC/TRF2DN-CM. Bars: 100 μm. **c**, CDK inhibitor expression in 3T3-L1 pre-adipocytes treated with the control medium, EC/GFP-CM or EC/TRF2DN-CM (n = 3 for control and EC/TERF2DN-CM; n = 4 for EC/GFP-CM). **d**, CDK inhibitor expression in C2C12 myotubes treated with the control medium, EC/GFP-CM or EC/TRF2DN-CM (n = 4 for control; n = 3 for EC-CM). A two-tailed Student's *t*-test was used for statistical analysis. Data are presented as mean ± s.e. $^{*}P < 0.05$.

Supplementary Fig. 4.

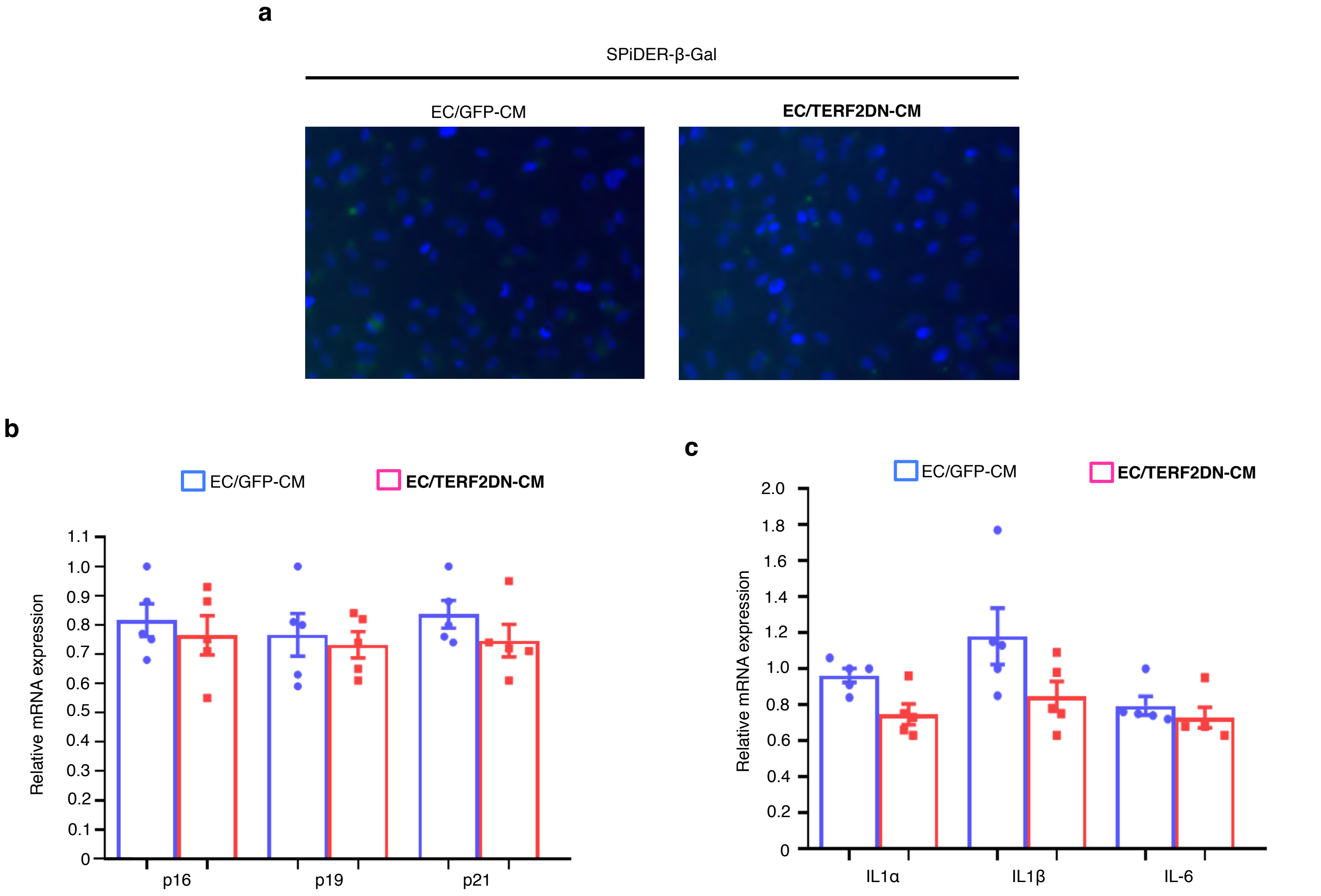

Fig. 4. Senescent EC does not induce senescence-like state in EC. a, SPiDER-β-Gal staining in ECs treated with CM derived from control ECs (EC/GFP-CM) or premature senescent ECs (EC/TERF2DN-CM). **b**, CDK inhibitor expression in ECs treated with the EC/GFP-CM or EC/TERF2DN-CM (n = 5 each). **c**, SASP factor expression in ECs treated with the EC/GFP-CM or EC/TERF2DN-CM (n = 5 each). A two-tailed Student's *t*-test was used for statistical analysis. Data are presented as mean ± s.e.

Supplementary Fig. 5

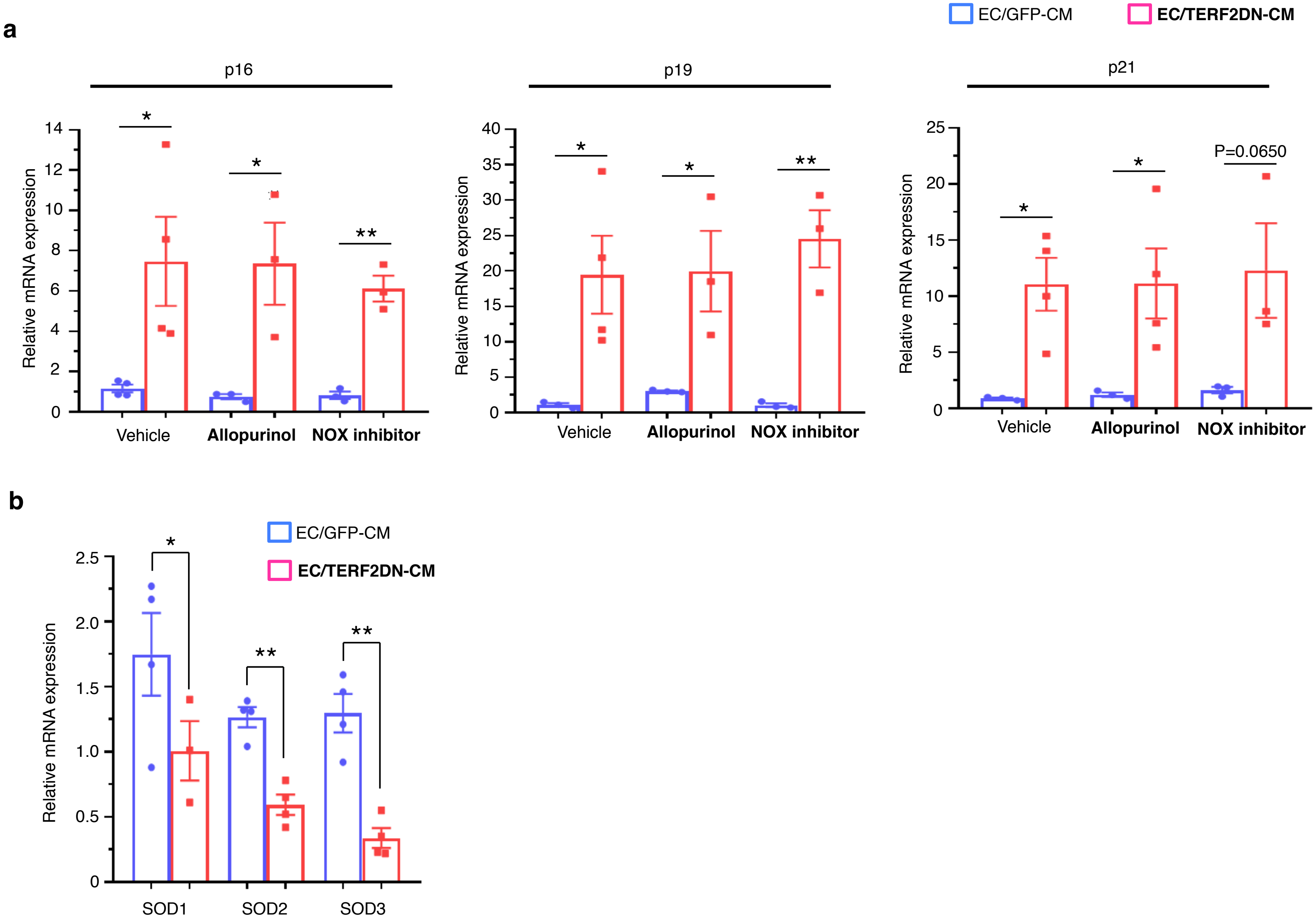

Fig. 5. Elimination of superoxide was impaired in adipocytes treated with senescent EC-CM. a, CDK inhibitor expression in 3T3-L1 adipocytes treated with the indicated EC-CM in the presence or absence of inhibitors for reactive oxygen species production by xanthine oxidase (allopurinol) or NADPH oxidase (NOX inhibitor) (n = 4 each for vehicle-treated cells; n = 3 each for inhibitor-treated cells). **b**, Superoxide dismutase (SOD) expression in 3T3-L1 adipocytes treated with the EC/GFP-CM or EC/TERF2DN-CM (n = 4 each). A two-tailed Student's *t*-test was used for statistical analysis. Data are presented as mean ± s.e. **P* < 0.05 and ***P* < 0.01.

Supplementary Fig. 6.

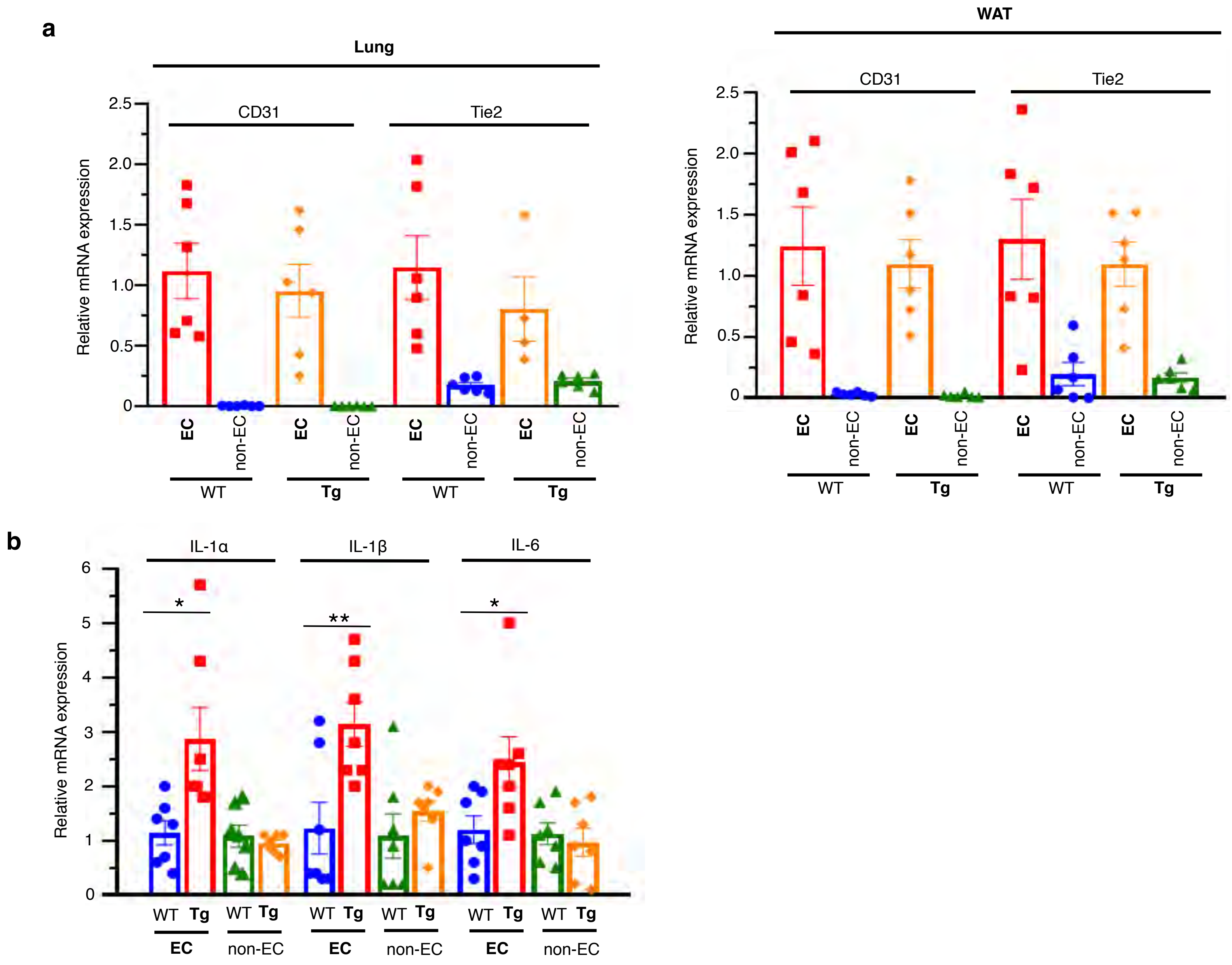

Fig. 6. Validation of EC-specific senescence in Tie2-TRF2DN-Tg mice. a, EC and non-EC were isolated from the lung and WAT of WT or Tie2-TRF2DN-Tg mice. EC marker expression was analyzed to confirm the successful EC isolation (n = 6 each). **b**, SASP factor expression in EC and non-EC isolated from the lung of WT or Tie2-TRF2DN-Tg mice (n = 7 each). A two-tailed Student's *t*-test was used for statistical analysis. Data are presented as mean ± s.e. *P < 0.05, **P < 0.01, and ****P < 0.0001.

Supplementary Fig. 7

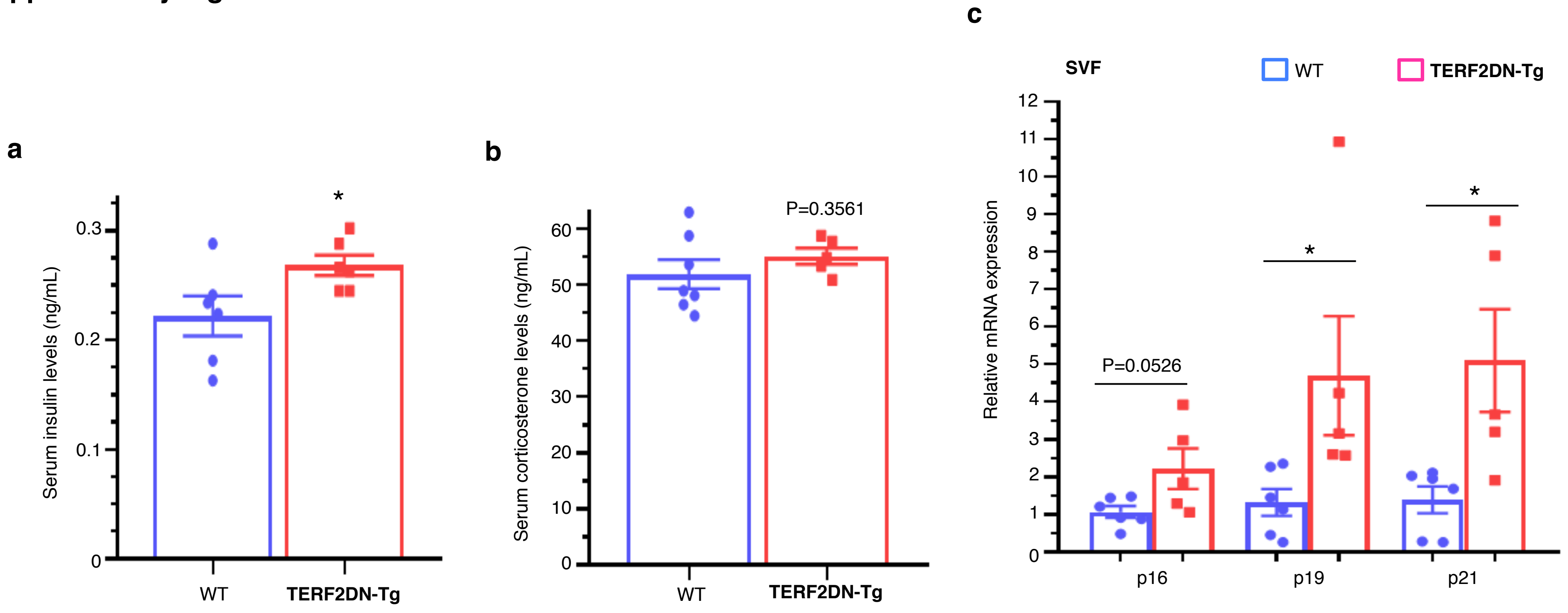

Fig. 7. Metabolic analysis for EC-specific progeroid mice at the age of 20 weeks old. a, Serum insulin levels in WT or Tie2-TERF2DN-Tg mice fed NC (n = 6 each). **b**, Serum corticosterone levels in WT or Tie2-TERF2DN-Tg mice fed NC (n = 7 for WT; n = 5 for Tg). **c**, CDK inhibitor expression in the stromal vascular fraction (SVF) isolated from the WAT of WT or Tie2-TERF2DN-Tg mice (n = 6 for WT; n = 5 for Tg). A two-tailed Student's *t*-test was used for statistical analysis. Data are presented as mean ± s.e. *P < 0.05.

Supplementary Fig. 8.

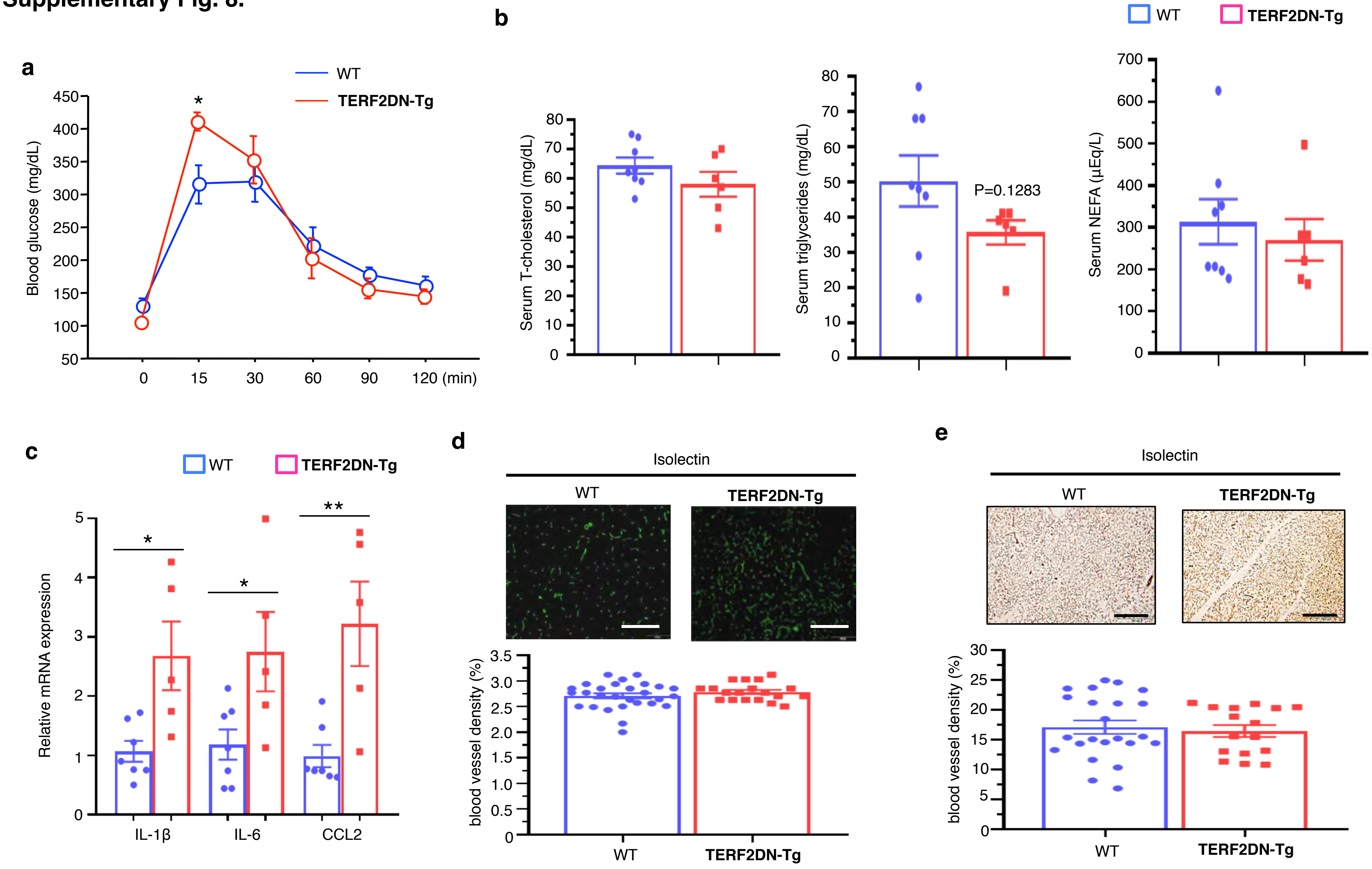

Fig. 8. Characterization of EC-specific progeroid mice at the age of 20 weeks old. a, Glucose tolerance test in 20-week-old WT and Tie2-TRF2DN-Tg mice fed normal chow (NC) (n = 7 for WT; n = 5 for Tg). **b**, Serum lipid profiles in WT or Tie2-TRF2DN-Tg mice fed NC at the age of 20-week old (n = 8 for WT; n = 6 for Tg). **c**, Inflammatory gene expression in the WAT of WT or Tie2-TRF2DN-Tg mice (n = 6 each). **d**, Capillary staining using isolectin in the WAT of WT (n = 28 fields) or Tie2-TRF2DN-Tg (n = 19 fields) mice. Bars: 100 μm. **e**, Capillary staining using isolectin in the BAT of WT (n = 23 fields) or Tie2-TRF2DN-Tg (n = 19 fields) mice. Bars: 200 μm. A two-tailed Student's *t*-test was used for statistical analysis. Data are presented as mean ± s.e. **P* < 0.05 and ***P* < 0.01.

Supplementary Fig. 9.

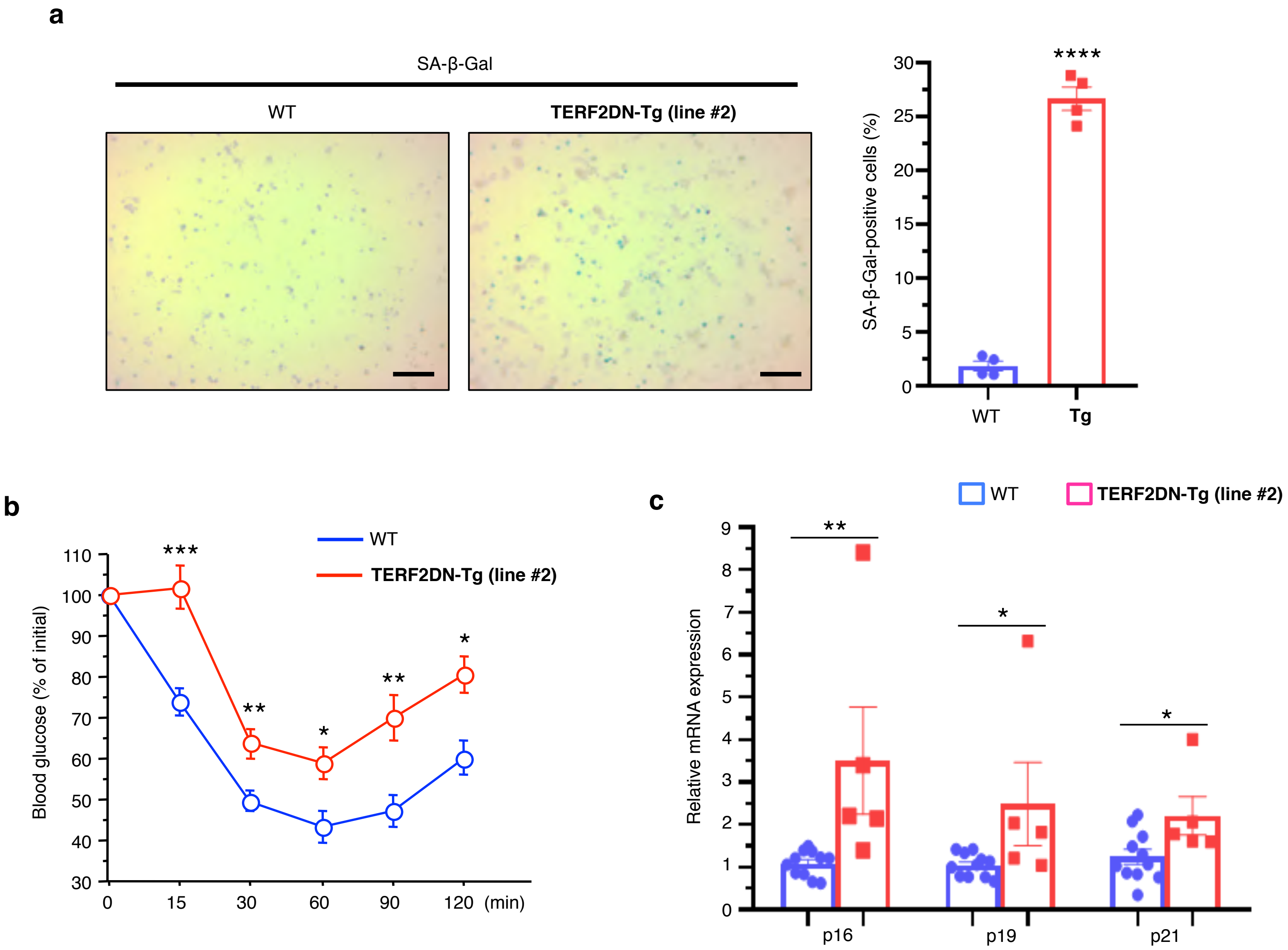

Fig. 9. Characterization of Tie2-TRF2DN-Tg (line #2) mice at the age of 20 weeks old. a, SA-β-Gal staining in ECs isolated from the lung of WT or Tie2-TRF2DN-Tg (line #2) mice. SA-β-Gal-positive cells were counted (n = 4 each). Bars: 100 µm. **b**, ITT in WT or Tie2-TRF2DN-Tg#2 mice fed NC at the age of 20 weeks old (n = 7 for WT; n = 5 for Tg). **c**, CDK inhibitor expressions in the WAT of WT (n = 11) or Tie2-TRF2DN-Tg#2 (n = 5) mice at the age of 20 weeks old. A two-tailed Student's *t*-test was used for statistical analysis. Data are presented as mean ± s.e. *$P < 0.05$, **$P < 0.01$, ***$P < 0.001$, and ****$P < 0.0001$.

Supplementary Fig. 10.

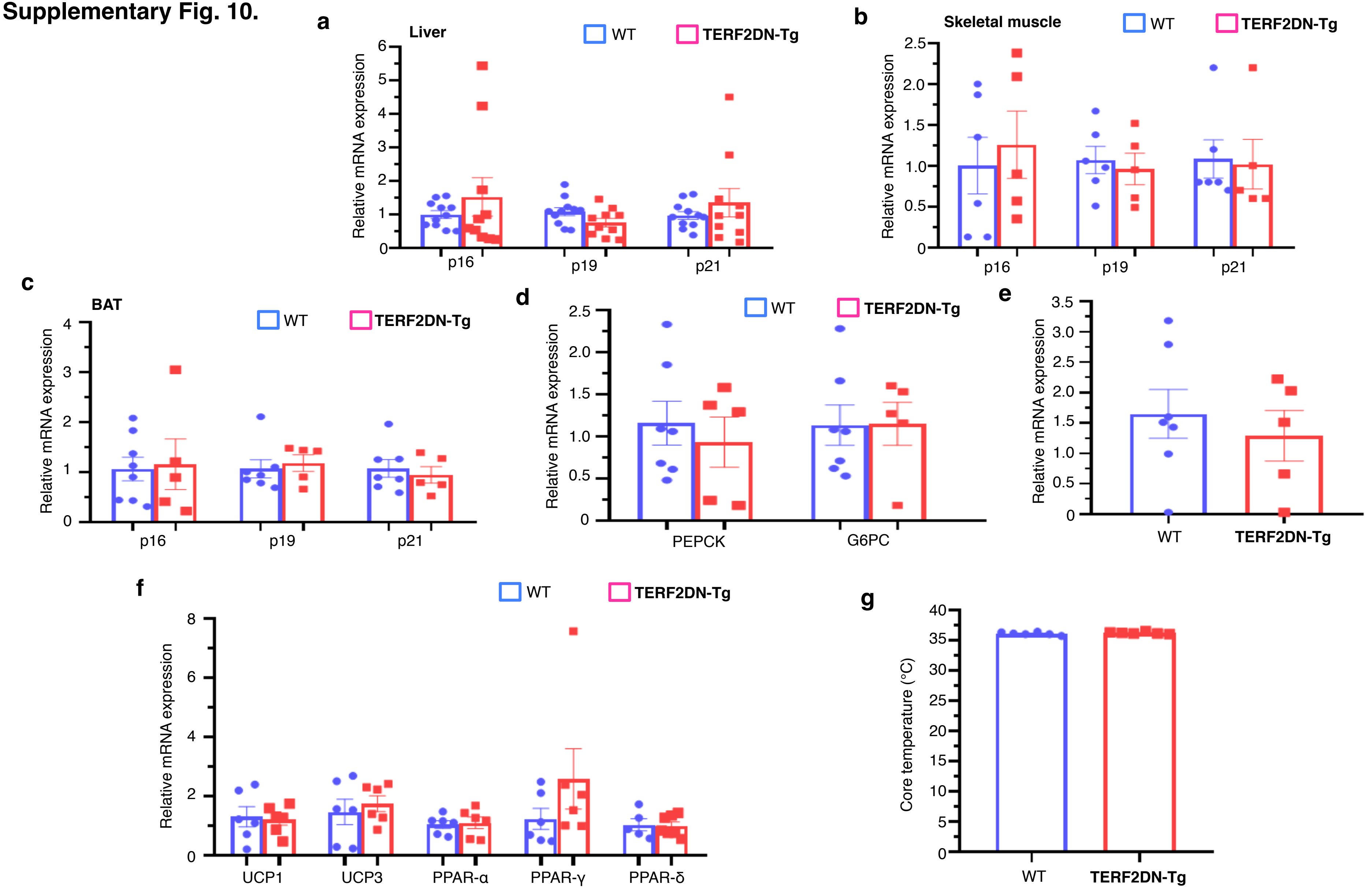

Fig. 10. Effect of EC progeria was minimal in the liver, skeletal muscle, and BAT. a, CDK inhibitor expression in the liver of WT or Tie2-TRF2DN-Tg mice at the age of 20 weeks old (n = 10 each). **b**, CDK inhibitor expression in the skeletal muscle of WT or Tie2-TRF2DN-Tg mice at the age of 20 weeks old (n = 6 for WT; n = 5 for Tg). **c**, CDK inhibitor expression in the BAT of WT or Tie2-TRF2DN-Tg mice at the age of 20 weeks old (n = 7 for WT; n = 5 for Tg). **d**, Phosphoenolpyruvate carboxykinase (PEPCK) and glucose-6-phosphatase (G6PC) expressions in the liver of WT or Tie2-TRF2DN-Tg (n = 7 for WT; n = 5 for Tg). **e**, PGC-1α expression in the skeletal muscle of WT or Tie2-TRF2DN-Tg mice (n = 7 for WT; n = 5 for Tg). **f**, UCPs and PPARs expressions in the BAT of WT or Tie2-TRF2DN-Tg mice (n = 7 each). **g**, Core temperature in WT or Tie2-TRF2DN-Tg mice under ambient condition (n = 6 each). A two-tailed Student's *t*-test was used for statistical analysis. Data are presented as mean ± s.e.

Supplementary Fig. 11.

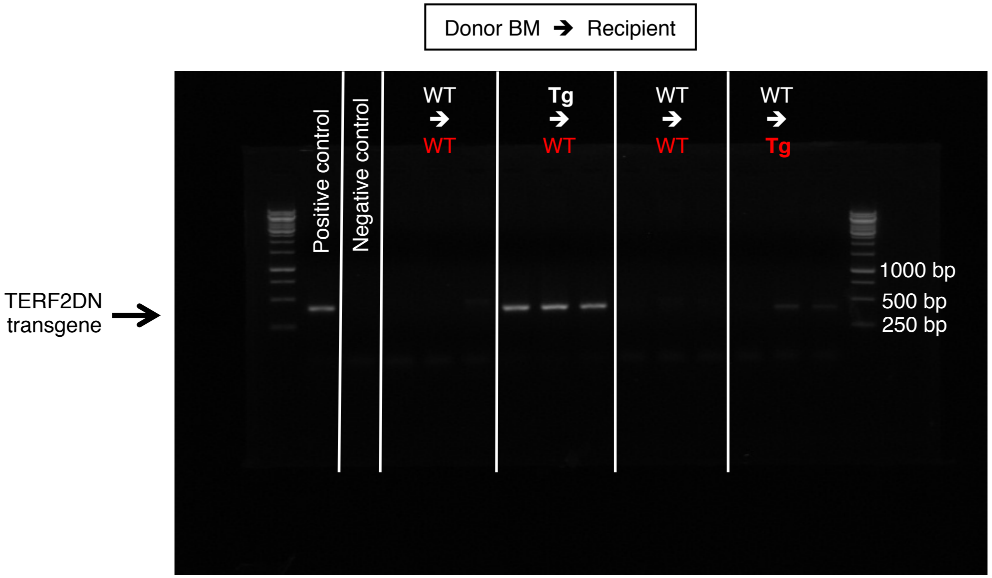

Fig. 11. Generation of BM-chimeric mice. Successful BM-transplantation was confirmed by detecting the transgene in BM cells isolated from each recipient mouse.

Supplementary Fig. 12.

a

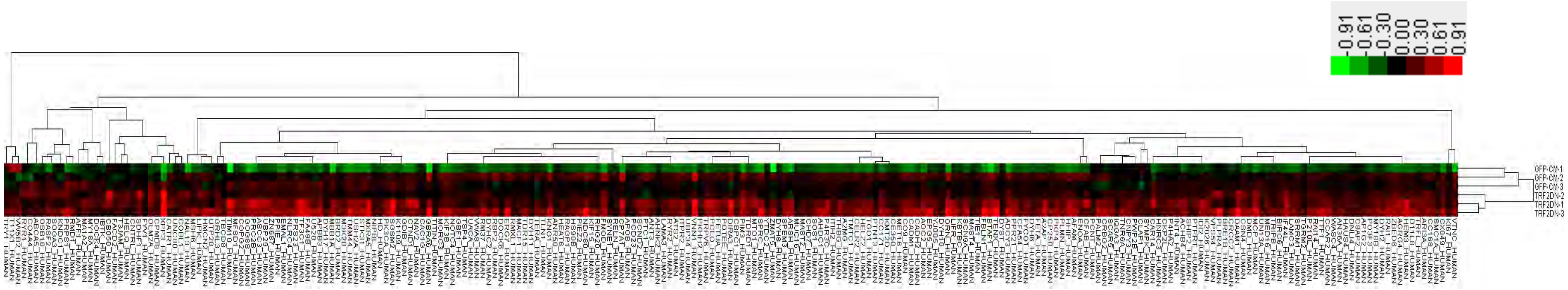

b

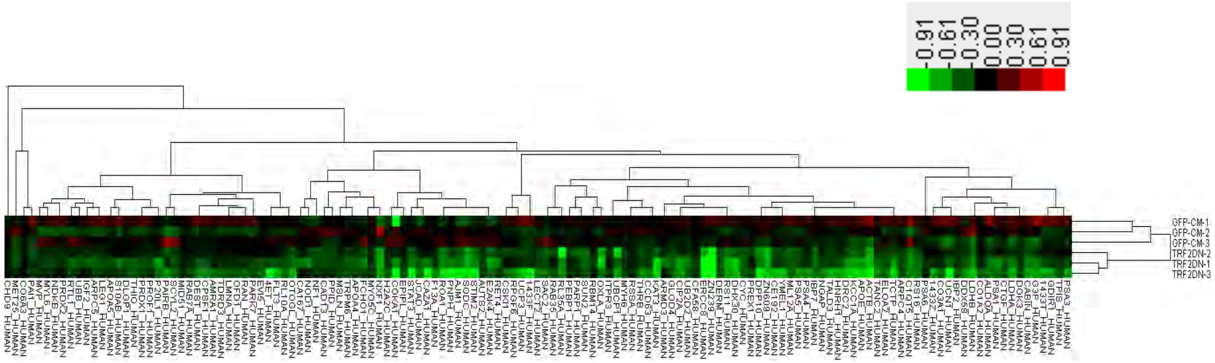

Fig. 12. Shotgun proteomics analysis for CM derived from young control or premature senescent EC. a, Heat map for proteins that were highly expressed in CM derived from premature senescent EC comparing with those in CM from young control EC ($P < 0.1$, n = 3 each). **b**, Heat map for proteins that were less expressed in CM from premature senescent EC than in CM from young control EC ($P < 0.1$, n = 3 each). Mann-Whitney U test was used for statistical analysis.

Supplementary Fig. 13.

Fig. 13. Pathway analysis for potential endothelial SASP factors. Most significant pathways identified among proteins that were increased in CM derived from premature senescent EC comparing with those in CM from young control EC were shown.

Pathway name	Entities				Reactions	
	found	ratio	p-value	FDR*	found	ratio
Regulation of Insulin-like Growth Factor (IGF) transport and uptake by Insulin-like Growth Factor Binding Proteins (IGFBPs)	10 / 124	0.011	4.55e-04	0.218	2 / 14	0.001
Post-translational protein phosphorylation	9 / 107	0.01	6.49e-04	0.218	1 / 1	8.19e-05
Platelet degranulation	9 / 128	0.011	0.002	0.38	2 / 11	9.01e-04
Extracellular matrix organization	15 / 301	0.027	0.003	0.38	74 / 318	0.026
Response to elevated platelet cytosolic Ca2+	9 / 133	0.012	0.003	0.38	2 / 14	0.001
Scavenging by Class H Receptors	2 / 4	3.59e-04	0.004	0.4	4 / 4	3.28e-04
ECM proteoglycans	6 / 76	0.007	0.007	0.454	9 / 23	0.002
Signaling by cytosolic FGFR1 fusion mutants	3 / 18	0.002	0.007	0.454	8 / 14	0.001
Signaling by PDGF	5 / 59	0.005	0.01	0.454	6 / 28	0.002
Non-integrin membrane-ECM interactions	5 / 59	0.005	0.01	0.454	4 / 22	0.002
Signaling by FGFR1 in disease	4 / 40	0.004	0.012	0.454	15 / 35	0.003
Intrinsic Pathway of Fibrin Clot Formation	3 / 22	0.002	0.013	0.454	8 / 20	0.002
Platelet activation, signaling and aggregation	12 / 262	0.023	0.013	0.454	25 / 114	0.009
Ligand-receptor interactions	2 / 8	7.17e-04	0.013	0.454	4 / 4	3.28e-04
Loss of proteins required for interphase microtubule organization from the centrosome	5 / 69	0.006	0.018	0.454	3 / 3	2.46e-04
Loss of Nlp from mitotic centrosomes	5 / 69	0.006	0.018	0.454	2 / 2	1.64e-04
Cohesin Loading onto Chromatin	2 / 10	8.97e-04	0.02	0.454	2 / 2	1.64e-04
Signaling by FGFR3 fusions in cancer	2 / 10	8.97e-04	0.02	0.454	4 / 9	7.37e-04
AURKA Activation by TPX2	5 / 72	0.006	0.022	0.454	2 / 2	1.64e-04
Interleukin receptor SHC signaling	3 / 27	0.002	0.022	0.454	3 / 6	4.92e-04
Type I hemidesmosome assembly	2 / 11	9.86e-04	0.024	0.454	4 / 6	4.92e-04
Signaling by FGFR4 in disease	2 / 11	9.86e-04	0.024	0.454	4 / 15	0.001
Laminin interactions	3 / 30	0.003	0.029	0.454	12 / 15	0.001
HSF1 activation	2 / 12	0.001	0.029	0.454	3 / 7	5.74e-04

Supplementary methods

Methods

Materials

Human umbilical vein endothelial cells (HUVECs) were purchased from Lonza. 3T3-L1 pre-adipocytes were obtained from JCRB cell bank in National Institutes of Biomedical Innovation, Health, and Nutrition. C2C12 cells were obtained from ATCC.

Antibodies for phospho-Akt (#9271), total-Akt (#9272), phospho-IRβ (#3024), total-IRβ (#3025), tri-methyl-histone H3 (Lys9) (H3K9me3) (#13696), phospho-Histone H2A.X (Ser139) (γH2AX) (#9718) and GAPDH (#2118) were purchased from Cell Signaling Technology. Antibodies for IRS-1 (#sc-559) and IRS-2 (#sc-1555) were purchased from Santa Cruz Biotechnology. Antibody for 8-OHdG (#MOG-020P) was purchased from JaICA. Antibody for Ki-67 (#418071) was purchased from Nichirei. Antibody forH3K9me2 (#39240) was purchased from Active Motif. Antibodies for FACS analysis including anti-IL-1a-PE (#130-104-481) and REA Control (I)-PE (#130-104-613) were purchased from Miltenyi Biotec.

The ELISA kit for mouse serum insulin measurements was purchased from Shibayagi. The ELISA kit for mouse corticosterone was purchased from Cayman Chemical. The ELISA kit for human IL-1a was purchased from R&D systems. Biotynilated Isolectin was obtained from Vector Labs and fluorescence-labeled isolectin GS-IB4 from Griffonia Simplicifolia was obtained from Invitrogen.

N-Acetyl-L-cysteine (NAC) was purchased from Sigma. β-Nicotinamide mononucleotide (β–NMN) was obtained from Oriental Bio. NOX inhibitor III was obtained from Calbiochem.

Allopurinol was obtained from SelleckChem. IL-1 receptor antagonist was obtained from WAKO. Hoechst 33342 was obtained from Molecular Probes.

Small interfering RNA (siRNA) for human IL-1a was obtained from Dharmacon (#M-007952-01-0005).

Immunoblotting

For immunoblotting, blots were incubated with antibodies for t-IRβ (1:1000), p-IRβ(1:1000), t-Akt (1:1000), p-Akt (1:1000), GAPDH (1:1000), IRS-1 (1:100), or IRS-2 (1:100) for overnight at 4 °C. Uncropped and unprocessed scans of the blots are provided in the Source Data file

Bone Marrow Transplantation

Bone marrow (BM) transplantation was performed as described previously[1]. Briefly, BM cells were isolated from Tie2-TRF2DN-Tg or WT mice, and 5×10^6 cells per body of BM cells were transfused into 10-week-old recipient mice that received 8 Gy lethal irradiation. Phenotypic analysis was performed 10 weeks after BM transplantation. Successful BM transplantation was confirmed by genotyping of BM cells and flow cytometry analysis of the reconstituted BM cells in control recipient WT mice transplanted with BM from GFP mice performed in a parallel way.

Metabolic measurements

The insulin and glucose tolerance tests (ITT and ipGTT) were performed as follows. For the ipGTT, mice fasted for 6 hours, and 1.5 g/kg D-glucose was intraperitoneally administered. For

the ITT, mice were given 1 IU/kg human insulin by subcutaneous injection without fasting. Blood glucose was measured by the glucose oxidase method (Johnson & Johnson K.K.). The adiposity of mice was examined and analyzed using computed tomography scanning (LaTheta LCT-100, Aloka) and its accompanied analysis software.

Insulin signaling study in vivo

Mice were given 1 IU/kg human insulin by subcutaneous injection without fasting. The visceral perigonadal WAT, soleus muscle, and liver were extracted 20 min after insulin-injection, followed by protein extraction and SDS-PAGE.

Isolation of mouse mature adipocytes and stromal vascular fraction from adipose tissue

Adipocytes and non-adipocytes were prepared using the collagenase method from epididymal white adipose tissue as previously reported[4]. Tissue homogenates were fractionated by brief centrifugation (350 x g for 20 s) in Krebs-Henseleit 4-(2-hydroxyethyl)-2-piperazine ethane sulfonic acid buffer, pH 7.4, supplemented with 20 mg/mL of bovine serum albumin (fraction V) and 2 M glucose. Floating cells were collected as mature adipocytes, and pelleted cells were used as the stromal vascular fraction. Subsequently, cells were homogenized with QIAzol lysis reagent (QIAGEN) for mature adipocytes or RNAiso Plus (TAKARA) for stromal vascular fraction.

Quantitative PCR

RNAs were purified using RNeasy Lipid Tissue Mini Kit (QIAGEN) for WAT, BAT and 3T3-L1 adipocytes, or using NucleoSpin RNA Clean-up kit (Macherey-Nagel) for the other types of cells and tissues according to the manufacturer's protocol. cDNA was synthesized from ~1 μg of total RNA using PrimeScript RT reagent Kit with gDNA Eraser (TAKARA). PCR reactions were prepared using FastStart SYBR Green Master (Roche Applied Science) followed by the real-time PCR analysis using LightCycler96 (Roche Applied Science). Quantification of gene expression was performed using the delta-delta CT method, which was normalized with 18S rRNA. Nucleotide sequences of the primers used are shown in Extended DataTable-1 and -2.

Histological analysis

Visceral perigonadal WAT was extracted and fixed with 4% paraformaldehyde, followed by paraffin embedding. Sections were prepared in 4 μm thickness. IHC for anti-8OHdG antibody or isolectin in the WAT sections was performed as previously reported[5]. After deparaffinization, sections were boiled in antigen unmasking solution (Vector Labs) for 10 min. Following the incubation in methanol containing 0.3% hydrogen peroxide for 15 min at room temperature, sections were incubated with 10% normal donkey serum in PBS-T (0.2% Triton-X in PBS). After blocking using avidin-biotin blocking kit (Vector Labs), sections were incubated with anti 8-OHdG antibody (1:200) or isolectin biotinylated (1:200) at 4 °C for overnight. Subsequently, sections were incubated with biotinylated secondary antibody (1:200) for 60 min at room temperature, followed ABC reaction (Vector Labs). Finally, immune-positive cells were visualized by incubating in DAB peroxidase substrate (Vector Labs). Isolectin-positive capillaries were quantified at 2–5 randomly chosen independent fields/each section.

Immunocytochemistry

For immunocytochemistry, cells were fixed with 4% PFA, and incubated with anti-Ki67 antibody (1:1, ready to use antibody), anti-H3K9me3 antibody (1:800) or anti-γH2AX (1:200) at 4°C for overnight. Following incubation with secondary antibodies labeled with alexa Fluor 594 (1:400), cells were covered with mounting medium for fluorescence with DAPI (Vector Labs), and observed under fluorescence microscope.

Superoxide detection

3T3-L1 adipocytes were treated with control medium or CM derived from young control or senescent ECs for 4 days, and then superoxide was detected using ROS/Superoxide Detection Kit (Enzo) as the manufacturer recommended. Nucleus was stained with Hoechst 33342.

Measurement of immunostain-positive areas

Immunostain-positive areas were measured using the NIH image software as previously reported[6,7]. Briefly, images of immunostained areas were extracted by the color-split function of the software, followed by quantification using the NIH image software.

***Ex vivo* cell isolation and sorting of endothelial cells from various murine organs**

Tissue dissociation and homogenization were performed using MACS dissociation kit (Miltenyi Biotec) for lung (#130-095-927) and WAT (#130-105-808); gentleMACS C tube

(#130-093-237); and gentleMACS Dissociators (#130-093-235) according the manufacturer's protocol. Following passing the MACS SmartStrainer (100 or 70 μm) and Pre-Separation Filter (30 μm), the dissociated cells were centrifuged at 300 x g for 10 min, and then resuspended in 90 μL of PEB buffer per 10^7 total cells. Cells were incubated with FcR Blocking reagent for mouse (#130-092-575) for 10 min at 4°C, followed by incubation with CD146 (LSEC) MicroBeads (#130-092-007) for 15 min at 4°C. After washing, cells were centrifuged at 300 g for 10 min, and then resuspended in 500 μL PEB buffer. Subsequently, cells were applied to LS column (#130-042-401) in the magnetic field of MACS separator (#130-042-301). The flow-through medium containing EC-depleted cells were collected, and these cells were used as non-ECs. After washing with PEB buffer for 3 times, isolated ECs were collected using 5 ml PEB buffer.

Shotgun proteomics analysis

Shotgun proteomeics analysis for conditioned medium derived from young control and premature senescent ECs were performed at Chemical Evaluations and Research Institute (CERI), Japan. Pathway analysis was performed by using the Reactome[8 9].

Flow cytometry

For fluorescent-activated cell sorter analysis, endothelial cells isolated from the lungs of 7-week-old or 70-week-old WT mice were stained with PE-labeled rat anti-IL-1a antibody (anti-IL-1a-PE, Miltenyi Biotec #130-104-481) or isotype control (REA Control (I)-PE, Miltenyi Biotec #130-104-613) in PBS containing 2% fetal calf serum. Cells were analyzed on a FACSCalibur (BD Bioscience) with FlowJo software (Tree Star). For elimination of cell debris,

cells were gated using forward and side scattered light, and were analyzed for cell-surface IL-1a expression levels. For each sample, 200,000 cells were analyzed. We calculated the mean fluorescence intensity and showed the fold change relative to the control group.

Supplementary Table

Table-1. Nucleotide sequence of mouse primers

MCP1 (CCL2)	GGCTCAGCCAGATGCAGTTAA
	CCTACTCATTGGGATCATCTTGCT
IL1α	CACATCAGCTGCTTATCCAGAGCTG
	GGTACATACAGACTGTCAGCACTTCC
IL6	ACAACCACGGCCTTCCCTACTT
	CACGATTTCCCAGAGAACATGTG
IL1β	CTGTGTCTTTCCCGTGGACC
	CAGCTCATATGGGTCCGACA
IL8 (CXCL-15)	CTGACCACTTAGCTTCTTCCTGACAG
	GACACAGTGTTCTTGCCTTGGCTCCA
p16 (Cdkn2a)	GACGGGCATAGCTTCAGCTCAAGCA
	GCCACATGCTAGACACGCTAGCATCGC
p19 (Cdkn2d)	GGAAGTCCAGCAGTTGCTTTGGAGCTC
	GGGCATTGACATCAGCACCATGCTCCA
p21 (Cdkn1a)	GCCACAGGCACCATGTCCAATCCTGG
	GCATCGCAATCACGGCGCAACTGCTC
18S	GTATAGGGCGCTCAAATGGA
	CCATCCAATCGGTAGTAGCCG
PEPCK	CGAAATTGAGAGGGAGCTCCGAGCC
	GATCTACTCAGCATTGTGCCGCTATCTC
G6PC	TGCTGGACCCTGCTGTGTCTGGTAGGC
	ATGAGAGCTCTTGGATGGCTTGGGCT
UCP-1	GGGCCCTTGTAAACAACAAA
	GTCGGTCCTTCCTTGGTGTA
UCP-3	CCACACTTCCTCCTGCTCTC
	GTAACCCGTTGAACCCCATT
PPAR-α	GAGGGTTGAGCTCAGTCAGG
	GGTCACCTACGAGTGGCATT
PPAR-γ	TTTTCAAGGGTGCCAGTTTC

	AATCCTTGGCCCTCTGAGAT
PPAR-δ	TGGAGCTCGATGACAGTGAC
	GTACTGGCTGTCAGGGTGGT
CD31	AACGAGAGCCACAGAGACGGTGTAC
	ATACGTGCACAGGACTCTCGCAATC
Tie2	AAGCATGCCCATCTGGTTAC
	GTAGGTAGTGGCCACCCAGA

Table-2. Nucleotide sequence of human primers

p16 (Cdkn2a)	CACCAGAGGCAGTAACCATGCCCGC
	GTAGGACCTTCGGTGACTGATGATC
p19 (Cdkn2d)	GGCAGTTCAAGAGGGTCACACTGCT
	ACCATGTGGCCCTGCAGGATGTCCA
P21 (Cdkn1a)	GGAAGACCATGTGGACCTGTCACTG
	AGATCAGCCGGCGTTTGGAGTGGTA
18S	GTAACCCGTTGAACCCCATT
	CCATCCAATCGGTAGTAGCG
MCP1 (CCL2)	GAAGAATCACCAGCAGCAAGTGTCCC
	GCTTGTCCAGGTGGTCCATGGAATCC
IL1α	GGTCACCAAATTCTACTTCCAGGAGGAC
	GTGACCAGGTTGTTGTGACGCCTTC
IL6	GAAGCTGCAGGCACAGAACCAGTGGC
	CTGACCAGAAGAAGGAATGCCCATT
IL1β	AGCTGTACCCAGAGAGTCCTGTGCTGA
	AGGAGAGAGCTGACTGTCCTGGCTGATG
IL8 (CXCL-15)	TCTGCAGCTCTGTGTGAAGGTGCAG
	GTGTGGTCCACTCTCAATCACTCTC

References for Supplementary Information

1 Matsuo, K. *et al.* Loss of apoptosis regulator through modulating IAP expression (ARIA) protects blood vessels from atherosclerosis. *The Journal of biological chemistry* **290**, 3784-3792, doi:10.1074/jbc.M114.605287 (2015).

2 Oike, Y. *et al.* Angiopoietin-related growth factor antagonizes obesity and insulin resistance. *Nat Med* **11**, 400-408, doi:10.1038/nm1214 (2005).

3 Tabata, M. *et al.* Angiopoietin-like protein 2 promotes chronic adipose tissue inflammation and obesity-related systemic insulin resistance. *Cell metabolism* **10**, 178-188, doi:10.1016/j.cmet.2009.08.003 (2009).

4 Kamei, Y. *et al.* Increased expression of DNA methyltransferase 3a in obese adipose tissue: studies with transgenic mice. *Obesity (Silver Spring)* **18**, 314-321, doi:10.1038/oby.2009.246 (2010).

5 Xue, Y., Lim, S., Brakenhielm, E. & Cao, Y. Adipose angiogenesis: quantitative methods to study microvessel growth, regression and remodeling in vivo. *Nature protocols* **5**, 912-920.

6 Jang, J. H., Rives, C. B. & Shea, L. D. Plasmid delivery in vivo from porous tissue-engineering scaffolds: transgene expression and cellular transfection. *Mol Ther* **12**, 475-483, doi:10.1016/j.ymthe.2005.03.036 (2005).

7 Goddard, J. C., Sutton, C. D., Furness, P. N., Kockelbergh, R. C. & O'Byrne, K. J. A computer image analysis system for microvessel density measurement in solid tumours. *Angiogenesis* **5**, 15-20 (2002).

8 Fabregat, A. *et al.* The Reactome Pathway Knowledgebase. *Nucleic Acids Res* **46**, D649-D655, doi:10.1093/nar/gkx1132 (2018).

9 Fabregat, A. *et al.* Reactome pathway analysis: a high-performance in-memory approach. *BMC Bioinformatics* **18**, 142, doi:10.1186/s12859-017-1559-2 (2017).
