# Supplementary material for: Endothelial progeria induces adipose tissue senescence and impairs insulin sensitivity through senescence associated secretory phenotype
Source: Nat Commun. 2020 Jan 24;11:481. doi: 10.1038/s41467-020-14387-w (PMC6981212; doi:10.1038/s41467-020-14387-w)
Supplement: Supplementary file 3 — Reporting Summary [file 41467_2020_14387_MOESM3_ESM.pdf]

nature research

Corresponding author(s): *Double-blind peer review submissions: write DBPR and your manuscript number here instead of author names.*

Last updated by author(s): *YYYY-MM-DD*

Reporting Summary

Nature Research wishes to improve the reproducibility of the work that we publish. This form provides structure for consistency and transparency in reporting. For further information on Nature Research policies, see Authors & Referees and the Editorial Policy Checklist .

Statistics

For all statistical analyses, confirm that the following items are present in the figure legend, table legend, main text, or Methods section.

n/a	Confirmed	
☐	☒	The exact sample size (*n*) for each experimental group/condition, given as a discrete number and unit of measurement
☐	☒	A statement on whether measurements were taken from distinct samples or whether the same sample was measured repeatedly
☐	☒	The statistical test(s) used AND whether they are one- or two-sided<br>*Only common tests should be described solely by name; describe more complex techniques in the Methods section.*
☐	☒	A description of all covariates tested
☐	☒	A description of any assumptions or corrections, such as tests of normality and adjustment for multiple comparisons
☐	☒	A full description of the statistical parameters including central tendency (e.g. means) or other basic estimates (e.g. regression coefficient) AND variation (e.g. standard deviation) or associated estimates of uncertainty (e.g. confidence intervals)
☐	☒	For null hypothesis testing, the test statistic (e.g. *F*, *t*, *r*) with confidence intervals, effect sizes, degrees of freedom and *P* value noted<br>*Give P values as exact values whenever suitable.*
☒	☐	For Bayesian analysis, information on the choice of priors and Markov chain Monte Carlo settings
☒	☐	For hierarchical and complex designs, identification of the appropriate level for tests and full reporting of outcomes
☒	☐	Estimates of effect sizes (e.g. Cohen's *d*, Pearson's *r*), indicating how they were calculated

Our web collection on statistics for biologists contains articles on many of the points above.

Software and code

Policy information about availability of computer code

Data collection	N/A
Data analysis	Statistical analysis was performed using the StatView 5.0 software.

For manuscripts utilizing custom algorithms or software that are central to the research but not yet described in published literature, software must be made available to editors/reviewers. We strongly encourage code deposition in a community repository (e.g. GitHub). See the Nature Research guidelines for submitting code & software for further information.

Data

Policy information about availability of data

All manuscripts must include a data availability statement. This statement should provide the following information, where applicable:

- Accession codes, unique identifiers, or web links for publicly available datasets
- A list of figures that have associated raw data
- A description of any restrictions on data availability

Source data are provided as a Source Data file.

Field-specific reporting

Please select the one below that is the best fit for your research. If you are not sure, read the appropriate sections before making your selection.

☒ Life sciences ☐ Behavioural & social sciences ☐ Ecological, evolutionary & environmental sciences

For a reference copy of the document with all sections, see nature.com/documents/nr-reporting-summary-flat.pdf

Life sciences study design

All studies must disclose on these points even when the disclosure is negative.

Sample size	We did not determine sample size statistically, but the sample size for each group typical for this type of work in literature was chosen.
Data exclusions	No data were excluded from the analysis.
Replication	All attempts at replication were successful.
Randomization	Randomization was used whenever possible.
Blinding	Investigators were not blinded to allocation during experiments and outcome assessment.

Reporting for specific materials, systems and methods

We require information from authors about some types of materials, experimental systems and methods used in many studies. Here, indicate whether each material, system or method listed is relevant to your study. If you are not sure if a list item applies to your research, read the appropriate section before selecting a response.

Materials & experimental systems

n/a	Involved in the study	
☐	☒	Antibodies
☐	☒	Eukaryotic cell lines
☒	☐	Palaeontology
☐	☒	Animals and other organisms
☒	☐	Human research participants
☒	☐	Clinical data

Methods

n/a	Involved in the study	
☒	☐	ChIP-seq
☐	☒	Flow cytometry
☒	☐	MRI-based neuroimaging

Antibodies

Antibodies used	p-IRb: CST #3024, t-IRb: CST #3025, p-Akt: CST #9271, t-Akt: CST #9272, IRS1: Santa Cruz #sc-559, IRS2: Santa Cruz #sc-1555, GAPDH: CST #2118, gH2AX: CST #9718, H3K9me3: CST #13696, H3K9me2: Active Motif #39240, Ki67: Nichirei #418071, IL1a-PE: Miltenyi Biotec #130-104-481, REA Control (I)-PE: Miltenyi Biotec #130-104-613, 8-OHdG: Japan Institute for the Control of Aging #MOG-020P
Validation	According to the data sheet, all the antibodies used for immunoblotting can detect target proteins. Also, 8-OHdG antibody is suitable for immunohistchemistry according to the data sheet.

Eukaryotic cell lines

Policy information about cell lines

Cell line source(s)	We used 3T3-L1 preadipocytes, and C2C12 myoblasts.
Authentication	3T3-L1 preadipocytes were provided by the cell bank of National Institutes of Biomedical Innovation, Health and Nutrition. C2C12 myoblasts were obtained from the ATCC.
Mycoplasma contamination	Culture cells were regularly tested for mycoplasma contamination. We have confirmed the negative results for the cells used in our experiments.
Commonly misidentified lines (See ICLAC register)	N/A

Animals and other organisms

Policy information about studies involving animals; ARRIVE guidelines recommended for reporting animal research

Laboratory animals	We have reported necessary information appropriately in the manuscript.
Wild animals	N/A
Field-collected samples	N/A

Ethics oversight	All animal experimental protocols were approved by the Ethics Review Committee for Animal Experimentation of Kobe Pharmaceutical University.

Note that full information on the approval of the study protocol must also be provided in the manuscript.

Flow Cytometry

Plots

Confirm that:

- [ ] The axis labels state the marker and fluorochrome used (e.g. CD4-FITC).
- [ ] The axis scales are clearly visible. Include numbers along axes only for bottom left plot of group (a 'group' is an analysis of identical markers).
- [ ] All plots are contour plots with outliers or pseudocolor plots.
- [x] A numerical value for number of cells or percentage (with statistics) is provided.

Methodology

Sample preparation	Endothelial cells were isolated from mouse lungs using MACS system (Miltenyi Biotec) according to the manufacturers protocols.
Instrument	FACSCalibur (BD Bioscience) was used for data collection.
Software	FlowJo software (Tree Star) was used to collect and analyze the data.
Cell population abundance	Successful endothelial cell isolation was confirmed by their abundant EC-markers expression as shown in the Supplementary Figure 6a.
Gating strategy	We described the gating strategy in the Supplementary Methods.

- [ ] Tick this box to confirm that a figure exemplifying the gating strategy is provided in the Supplementary Information.
